# Paraoxonases (PON) 1, 2, and 3 Polymorphisms and PON-1 Activities in Patients with Sickle Cell Disease

**DOI:** 10.3390/antiox8080252

**Published:** 2019-07-30

**Authors:** Cadiele Oliana Reichert, Carolina Garcia de Macedo, Débora Levy, Bruno Carnevale Sini, Andréia Moreira Monteiro, Magnus Gidlund, Luciana Morganti Ferreira Maselli, Sandra Fátima Menosi Gualandro, Sérgio Paulo Bydlowski

**Affiliations:** 1 Lipids, Oxidation, and Cell Biology Group, Laboratory of Immunology (LIM19), Heart Institute (InCor), Hospital das Clínicas HCFMUSP, Faculdade de Medicina, Universidade de Sao Paulo, 05403-900 Sao Paulo, Brazil; 2 Department of Immunology, Institute of Biomedical Sciences, Universidade de Sao Paulo, 05508-000 Sao Paulo, Brazil; 3 Department of Hematology, Faculdade de Medicina FMUSP, Universidade de Sao Paulo, 05419-000 Sao Paulo, Brazil; 4 Center of Innovation and Translational Medicine (CIMTRA), Department of Medicine, Faculdade de Medicina FMUSP, Universidade de Sao Paulo, 05360-130 Sao Paulo, Brazil; 5 Instituto Nacional de Ciencia e Tecnologia em Medicina Regenerativa (INCT-Regenera), CNPq, 21941-902 Rio de Janeiro, Brazil

**Keywords:** paraoxonase, sickle cell disease, PON-1, ferritin, transferrin, polymorphism, oxidized cholesterol

## Abstract

(1) Background: Oxidative stress, chronic inflammation, vasoocclusion, and free iron are all features present in sickle cell disease. Paraoxonases (PON) are a family (PON-1, PON-2, PON-3) of antioxidant enzymes with anti-inflammatory action. Here, for the first time, we described PON-1 activities and PON-1, PON-2, PON-3 polymorphisms in patients with sickle cell disease, homozygous for HbSS, compared with healthy controls. (2) Methods: The groups were matched for age and gender. PON-1 activities (arylesterase and paraoxonase) were determined by enzymatic hydrolysis of phenylcetate and paraoxon, respectively. Polymorphisms were determined by Restriction Fragment Length Polymorphism- Polymerase Chain Reaction (RFLP-PCR). (3) Results: Plasma cholesterol and fractions, ApoA1 and ApoB levels were all decreased in sickle cell disease patients, while anti-oxidized low-density lipoprotein (LDL) antibodies and C-reactive protein were increased. Serum arylesterase activity was lower in sickle cell disease patients when compared with healthy controls. In patients, paraoxonase activity was higher in those with PON-1 RR Q192R polymorphism. In these patients, the increase of serum iron and ferritin levels and transferrin saturation were less pronounced than those observed in patients with QQ or QR polymorphism. No differences were observed with PON-1 L55M, and PON-2 and PON-3 polymorphisms. Multivariate regression analysis showed that transferrin and ferritin concentrations correlated with arylesterase and paraoxonase activities. (4) Conclusions: Both transferrin and ferritin were the main predictors of decreased arylesterase and paraoxonase activities in patients with sickle cell disease. LDL oxidation increased, and RR PON-1 Q192R polymorphism is likely to be a protective factor against oxidative damage in these patients.

## 1. Introduction

Sickle cell disease (SCD) is a monogenic disorder caused by a point mutation in the sixth codon of exon 1 in chromosome 11, with a substitution of an adenine by a thymine nucleotide (GAG → GTG), resulting in the codification of valine instead of glutamic acid in the sixth position of hemoglobin β chain (β6Glu → Val). Sickle cell anemia (SCA) represents the homozygous condition of the βS globin allele (HbS) [1]. Phenotypically, this alteration causes hemoglobin to polymerize within erythrocytes during deoxygenation, altering red blood cell rheology and causing hemolysis. Erythrocytes containing intracellular hemoglobin polymer are less deformable [2]. They become entrapped within the microcirculation leading to tissue ischemia, reperfusion injury, and infarction [3].

The chronic effects of maintained hemolytic anemia and vaso-occlusive events in turn lead to the development of end-organ complications resulting in several events, such as pulmonary hypertension, left ventricular diastolic heart disease, arrhythmias, renal disease, neurological and hepatic complications, and sudden death [3,4]. Moreover, the chronic effect of sustained hemolytic anemia derived from the fragility of erythrocyte membranes and iron overload, is entailed by the release of the heme group during intravascular hemolysis [3,4,5]. In the process of hemolysis, the released Fe^3+^ is highly reactive, being deposited in tissues, oxidizing cell membranes, lipids, lipoproteins, and increase production of reactive oxygen species (ROS) and free radicals [6,7]. Higher amounts of ROS and free iron, through Fenton and Haber–Weiss reactions, can lead to changes in the activity of some antioxidant enzymes, such as paraoxonases [8].

Paraoxonases (PONs) are a multigene family of enzymes composed of three members: PON-1, PON-2, and PON-3, located adjacent to human chromosome 7 (7q21–23) [9]. The study of paraoxonases has been of great interest due to their role on oxidative stress and its anti-inflammatory activity. Currently, it is known that PON-1 and PON-3 are bound to high density lipoprotein (HDL), and PON-2 is an intracellular enzyme located in the mitochondria and endoplasmic reticulum [10,11,12]. PON-1 binds to HDL through their N-terminal hydrophobic tails and, in a lesser extent, can associate with (very-low density lipoprotein) VLDL and postprandial chylomicrons [13]. In this way, PON-1 can be transported to tissues in order to exert its antioxidant function [13]. Therefore, HDL has an anti-inflammatory effect and antioxidant properties that may prevent the oxidation of low-density lipoprotein (LDL). Oxidized LDL (oxLDL) increases the risk of both endothelial dysfunction and changes in vessels owing to infiltration by leukocytes and induction of inflammation [8,14,15,16].

PON-1 has paraoxonase, arylesterase, and lactonase activities, defined by both environmental and genetic factors [11,17]. There are two isoforms of PON-1, one due to substitution of amino acid glutamine (Gln) for arginine (Arg) (Q → R) at position 192, the other due to the substitution of a leucine (Leu) by methionine (Met) (L → M) at position 55 [9]. These polymorphisms have been associated with PON-1 activity [18]. However, enzymatic activity and the distribution of the genotypic frequency of PON-1, PON-2, and PON-3 were demonstrated to be different depending on the population and diseases involved. In this way, PON-1 activities may be associated with pathophysiological events of different diseases, such as coronary atherosclerosis, peripheral insulin resistance, metabolic syndrome, neurological disorders, as well as infectious diseases [12,17,19,20,21,22].

Here, for the first time, the arylesterase and paraoxonase activities of PON-1 were evaluated, as well as the frequency of the polymorphism in PON-1, PON-2, and PON-3 genes, in patients with sickle cell disease.

## 2. Materials and Methods

### 2.1. Study Design and Participants

This study was approved by the Ethics Committee of the Institution (HCFMUSP) (research protocol n° 0285/10). All participants gave their written informed consent. This study included 43 patients with sickle cell disease in a stable (steady) state. All patients were homozygous for HbSS. The selection criteria for individuals with sickle cell disease were: (I) not be under pharmacological treatment with hydroxyurea, (II) no recent episodes of pain crises (last three months), (III) no recent blood transfusion (last three months), and (IV) be at least 18 years old.

The control group consisted of 43 healthy volunteers with no clinical or analytical evidence of renal insufficiency, liver or neurological disease, neoplasia, chronic infection and inflammation, dyslipidemia, and blood transfusion.

### 2.2. Sample Preparation

Venous blood samples (15 mL) were collected after 8–12 h fasting using a vacuum system (Vaccutainer ^®^) with and without EDTA. Blood without EDTA was immediately centrifuged at 420× *g* for 15 min and serum was stored at −80 °C until use. The blood collected with EDTA was used for genomic DNA extraction from leukocytes by the salting out precipitation method [23].

### 2.3. Biochemical Analyses

The concentrations of serum total cholesterol (TC) as well as their fractions (HDL, LDL, and VLDL), triglycerides (TG), apolipoprotein (Apo) A1 and B, C-reactive protein (CRP), total, direct and indirect bilirubin, lactate dehydrogenase, fibrinogen, hematological parameters, and iron profile were measured. Serum markers for iron and lipids were measured by standard methods in a Modular 48 Analytics P-800 (Roche Diagnostics Corporation, Indianopolis, IN, USA) automated analyzer. A modified Clauss method was also used for fibrinogen determination [24]. Ultrasensitive C-reactive protein (us-CRP) was assessed by immunoturbidimetric assay. Anti-oxLDL antibodies were determined as a marker of oxidative stress according to the methodology described by Fernvik et al. [25] and Brandão et al. [26].

### 2.4. PON-1, PON-2, and PON-3 Genotypes

Analysis of the PON-1 (Q192R and L55M) polymorphisms was performed using primers designed in order to introduce a recognition site for *Hinf I* enzyme in one allele of each PCR product. This strategy allowed simultaneous identification of two polymorphisms of PON-1 in a single assay amplification followed by restriction analysis [27]. Alelles Q192 and 192R were assigned based on the presence of a 111 bp (undigested) fragment, and 77 and 34 bp (digested) fragments. Alelles L and M for the 55 position were assigned based on the presence of 144 bp (undigested) fragment, and 122 and 22 bp (digested) fragments. Genotyping of codon A148G of PON-2 was performed by PCR, as described by Hegele et al. [28]. The S311C polymorphism was determined according to method described by Motti et al. [27]. Primers sequences are given in Table 1. The samples were analyzed on agarose gel to 4%. The polymorphisms G10340T, A2115T, A45486C, and 55146CT in the PON-3 gene were analyzed by real-time PCR according to the TaqMan SNP Genotyping Assay Protocol from Applied Biosystems. PCR Universal TaqMan Master Mix (concentration 2×); Primers and Probes FAM/VIC (concentration 20×). The single nucleotide polymorphism (SNPs) TaqMan used in this study were: C_11708898_10 (T10340G), C_59001595_10 (C45486A), C_59001773_10 (A2115T), e C_59001534_10 (C55146T) (7500 fast real-time PCR system Applied Biosystems).

### 2.5. PON-1 Activities

The determination of the arylesterase activity of PON-1 was performed as described by Eckerson et al., [18] based on phenylcetate hydrolysis. The reaction kinetics of phenol formation was monitored with a spectrophotometer at 25 °C in a wavelength of 270 nm at intervals of 30 s for 5 min. Results were expressed as U/mL

The paraoxonase activity of PON-1 was determined according to Senti et al. [29] and Agachan et al. [30]. The enzymatic hydrolysis of paraoxon releases *P*-nitrophenol, whose rate of formation was evaluated spectrophotometrically with absorbance readings at 405 nm at 37 °C for 10 min.

### 2.6. Statistical Data Analysis

The Shapiro–Wilk test was used to determine the characteristics of data distribution. Both groups (sickle cell disease and healthy control) were matched for age and sex. Results were shown as mean ± standard deviation (SD). Student’s t-test and the non-parametric Mann–Whitney U test were used to compare the quantitative variables between the groups. The genotype frequencies of the polymorphisms were calculated using the Hardy–Weinberg Equilibrium (HWE), Chi-square test (χ^2^), and Fisher’s exact test. Pearson correlation analysis was performed between the PON-1 activities and all other data. Multivariate linear regression was used to establish the relationship between iron markers and PON-1 activities. *P* ≤ 0.05 values were considered as significant. SPSS software (IBM SPSS version 22.0) was used for all analyses.

## 3. Results

### 3.1. Gender and Age of Subjects from Sickle Cell Disease and Healthy Control Groups

The population of this study consisted of 43 patients with sickle cell disease and 43 healthy individuals matched for age and sex. There was no significant difference in the distribution of male or female participants between the two groups (Table 2). The percentage of women was higher than that of men in both groups.

### 3.2. Cholesterol and Fractions, Apo-A1 and B in Patients with Sickle Cell Disease and Healthy Controls

The results are shown in Table 3. Total cholesterol was reduced in the patient group when compared to healthy controls. Moreover, cholesterol was also reduced in both HDL and LDL (HDL-C and LDL-C) in sickle cell disease, whereas triglyceride and VLDL-C levels did not change. Apo-A1 and Apo-B levels were decreased in the patient group, although the ApoB:ApoAI ratio did not change.

### 3.3. PON-1 Activities in Sickle Cell Disease Patients and Healthy Controls

Although both arylesterase and paraoxonase PON-1 activities were reduced in patients with sickle cell disease compared with healthy controls (Table 3), only arylesterase activity reached the significance level.

Arylesterase activity in the sickle cell disease group was positively correlated with total cholesterol (r = 0.407; *P* = 0.01), HDL-C (r = 0.334; *P* = 0.01), LDL-C (r = 0.270; *P* = 0.01), Apo-A1 (r = 0.427; *P* = 0.01), and Apo-B (r = 0.170; *P* = 0.01) concentrations.

A positive correlation of paraoxonase activity with HDL-C (r = 0.296; *P* = 0.01) and Apo-A1 (r = 0.259; *P* = 0.01) levels was observed in patients with sickle cell disease.

High serum concentration of ultrasensitive C-reactive protein correlated negatively with both arylesterase (r = −0.179; *P* = 0.001) and paraoxonase activities (r = −0.229; *P* = 0.001) in the sickle cell disease group.

The levels of anti-oxLDL antibodies were higher in patients with sickle cell disease when compared with the healthy control group (Table 3) and was negatively correlated with arylesterase activity (r = −0.107; *P* = 0.001).

### 3.4. Transferrin and Ferritin as Predictors for PON-1 Arylesterase and Paraoxonase Activities in Patients with Sickle Cell Disease

All iron metabolism markers were evaluated as predictors of the PON-1 arylesterase and paroxonase activities with the regression model (Table 4). Serum ferritin concentration was not a predictor to PON-1 arylesterase (R^2^ = 0.047; *P* = 0.277) or paraoxonase (R^2^ = 0.000; *P* = 0.923) activities. However, statistical significance was achieved with the addition, to the regression model, of serum transferrin as a predictor of the arylesterase (R^2^ = 0.256; *P* = 0.032) or paraoxonase (R^2^ = 0.316; *P* = 0.011) activities. Addition of transferrin saturation or serum iron did not change the prediction for arylesterase (R^2^ = 0.266; *P* = 0.064 and R^2^ = 0.332; *P* = 0.055, respectively) but was significant for paraoxonase (R^2^ = 0.316; *P* = 0.030 and R^2^ = 0.378; *P* = 0.028, respectively). Addition of Total Iron Binding Capacity (TIBC) was not significant for either, arylesterase or paraoxonase (R^2^ =0.367; *P* =0.069 and R^2^ = 0.385; *P* = 0.054, respectively).

### 3.5. Other Biochemical Determinations

Other biochemical parameters were analyzed as possible predictors for PON-1 arylesterase and paraoxonase activities using the multivariate linear regression analysis (Table 4). In patients with sickle cell disease, there was an increase in the concentration of lactate dehydrogenase, total bilirubin, indirect bilirubin, and reticulocyte percentage. These results are characteristic of intravascular hemolysis in sickle cell disease. However, no relation and/or association between those hemolysis markers and arylesterase and paraoxonase activities were observed.

The allele distribution of the PON-1, PON-2, and PON-3 polymorphisms followed the Hardy–Weinberg equilibrium in the sickle cell disease group as well as in the healthy control group.

The PON-1 Q192R and R192R polymorphism frequencies were higher in the sickle cell disease group, whereas Q192Q genotype frequency was higher in the healthy control group (Table 5). The frequency of Q allele was higher in the healthy control group, and the R allele frequency was higher in the sickle cell anemia group.

The biochemical and hematological parameters were also evaluated in relation to the PON-1 Q192R polymorphism in sickle cell disease patients (QQ, QR, and RR phenotypes). The percentage of reticulocytes was higher in the QQ group (15.36 ± 6.37) when compared with the RR group (10.33 ± 3.95). Patients with the RR phenotype showed a decreased ferritin concentration (211.16 ± 138.50 ng/dL), transferrin saturation (35.17 ± 13.65%), and iron concentration (99.46 ± 41.30 µg/dL) when compared with the group with the QQ phenotype (1,324.14 ± 1,823.66 ng/dL, 53.17 ± 20.99%, and 147.14 ± 50.97 µg/dL, respectively). Although these data might indicate that the PON-1 QQ phenotype could be related to a more severe form of sickle cell anemia, more studies should be performed. No differences were observed in the other measured parameters.

No differences were observed in genotype distributions and relative frequencies of alleles of the PON-1 L55M polymorphism, and in all studied PON-2 and PON-3 polymorphisms between both groups (Table 5).

### 3.6. PON-1 Arylesterase and Paraoxonase Activities

**Figure 1:**
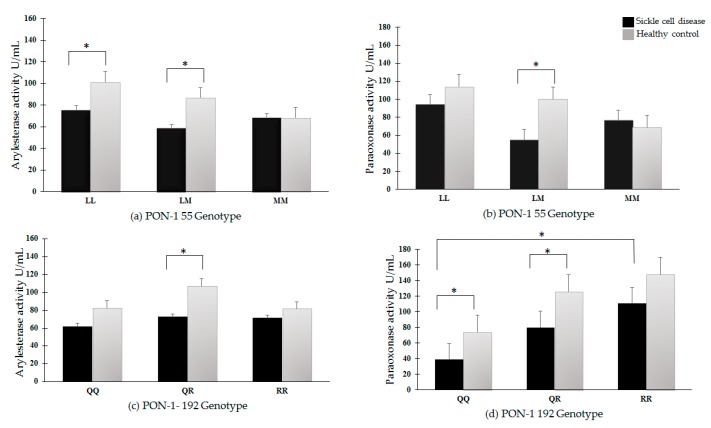
Serum PON-1 arylesterase and paraoxonase activities in subjects with PON-1 L55M and Q192R polymorphisms. (**a**) Arylesterase activity and PON-1 L55M polymorphisms; (**b**) Paraoxonase activity and PON-1 L55M polymorphisms; (**c**) Arylesterase activity and PON-1 Q192R polymorphisms; (**d**) Paraoxonase activity and PON-1 Q192R polymorphisms. * *P* < 0.05.

The arylesterase and paraoxonase activities of PON-1 were evaluated in relation to the determined PON-1 polymorphisms (Figure 1).

Regarding the PON-1 L55M polymorphism, the arylesterase activity was lower in LL and LM patients with sickle cell disease compared with healthy individuals, while it was not changed in MM patients (Figure 1a). The paraoxonase activity was lower in LM patients when compared to healthy subjects, while it did not change in LL or MM patients (Figure 1b).

Examining the PON-1 Q192R polymorphism, however, the arylesterase activity was lower only in QR sickle cell disease patients, compared with healthy subjects (Figure 1c). Paraoxonase activity was lower in QQ and QR sickle cell disease patients, while it did not change in RR patients (Figure 1d). It is noteworthy that the value of paraoxonase activity in patients with RR polymorphism was almost double in relation to those with QQ polymorphism (Figure 1d). The same was observed in healthy individuals, although no significance has been observed.

## 4. Discussion

Paraoxonases 1, 2, and 3 polymorphisms, and PON-1 activities were evaluated in patients with sickle cell disease. Hemolysis, vasoocclusion, oxidative stress, and chronic inflammation, are all hallmarks in sickle cell disease, in which iron overload is a feature [1,31,32]. The antioxidant capacity of PON-1 is attributed to its arylesterase, lactonase, and paraoxonase activities. PON-1 functions as a peroxidase, leading to the neutralization of fatty acids, cholesteryl ester hydroperoxides, and hydrogen peroxides [17]. The inflammation process as well as hydroxyl radicals, reactive oxygen species, and oxidizing agents, affect PON-1 activities [17,22,33].

In this study, total cholesterol, HDL-C, LDL-C, Apo-A1, and Apo-B, as well arylesterase PON-1 activity were decreased in sickle cell disease patients. PON-1 binds to HDL through the interaction with Apo-A1 and phospholipids, with Apo-A1 being responsible for the stabilization of PON-1 [34]. Serum alterations in Apo-A1 can directly interfere with PON-1 activities [35]. In fact, in chronic inflammatory process, HDL is subjected to modifications that include the reduction in the content of PON-1 and oxidation of its structure [36]. Therefore, the decreased PON-1 activities in patients with sickle cell disease observed in this study may be due, at least in part, to alterations in HDL and apo-A1 structures. Besides, oxidative stress is known to also be caused by the increase of free iron in diseases with iron overload [36]. Moreover, free iron can alter the function of Apo-A1 [37]. Therefore, these events may directly interfere in the decrease of arylesterase activity observed in patients. oxLDL is a marker of oxidative stress [38]. The increased plasma anti-oxLDL antibodies observed in patients with sickle cell disease could reflect the decreased HDL capacity to protect LDL against oxidation.

Transferrin is responsible for the transport of ferric iron in the body and ferritin for the storage of iron [39,40]. Besides, transferrin and ferritin are also acute phase proteins. The decrease of PON-1 activities has been associated with serum ferritin and transferrin in several diseases, such as Parkinson disease [41], inflammatory bowel disease [42], iron deficiency anemia [43], hereditary hemochromatosis with iron overload [36], and in beta-thalassemia [44,45]. Here, the increased levels of transferrin and ferritin were the main predictors of arylesterase and paraoxonase activities in the patients with sickle cell disease, both being responsible for approximately 25% of the arylesterase activity and 31% of the paraoxonase activity. It is tempting to postulate that both transferrin and ferritin could be a link between the inflammatory process underlying sickle cell disease and serum PON-1 activities.

Increased concentrations of lactate dehydrogenase and C-reactive protein are particularly interesting, since the first is increased in endothelial dysfunction, vascular obstruction, and intravascular hemolysis [1,2,7], and the high levels of the second has been associated with vaso-occlusive events [38]. In this study a negative correlation was found between PON-1 activities and C-reactive protein in the sickle cell disease group, whereas no relationship was seen between PON-1 activities and lactate dehydrogenase or any other increased markers of hemolysis or inflammation. The importance of these findings remains to be investigated.

The higher frequency of the RR homozygotes of the PON-1 Q192R polymorphism and the low frequency of the QQ and QR genotypes in patients with sickle cell disease, compared with healthy controls, were unexpected. The RR genotype has been associated with protection against oxidative stress [46] while the QQ genotype has been related to a decrease in protection against oxidation of HDL-C and LDL-C and increased DNA damage by organophosphates and hydroxyl radicals [47,48]. Our results reinforce these findings, and the higher paraoxonase activity was associated with the homozygous RR genotype in patients with sickle cell disease. Intriguingly, the RR genotype has been associated with the binding of PON-1 to HDL. In serum from individuals with RR homozygous genotype a strong link between PON-1 and HDL-Apo-AI was found, whereas the QQ and RQ genotypes were related to free PON-1 fractions [49,50].

Moreover, in this study, sickle cell disease patients with PON-1 RR polymorphism had a lower serum ferritin and iron concentration and transferrin saturation when compared with those with QQ or QR polymorphisms. Transferrin saturation was described to increase the risk of iron and/or free iron (non-transferrin-bound iron) extrahepatic deposition [51]. Free iron increases ROS production through the Haber–Weiss reaction, mainly OH radicals, leading to several features, such as increased lipid peroxidation in hepatic lysosomal membranes, or damage of cardiac mitochondria [52]. In patients with sickle cell disease, the increase in both ferritin and transferrin saturation has been associated with iron overload, hepatic, myocardial and endocrine organs dysfunctions, as well as severity, morbity, and mortality of the disease [3,53,54,55].

Therefore, the increase in paraoxonase activities and less pronounced increase of serum ferritin and iron concentrations and transferrin saturation, all related to RR polymorphism when compared with QR and RR polymorphisms, may contribute to protection against the damage caused by oxidative stress iron in patients with sickle cell disease.

## 5. Conclusions

In conclusion, patients with sickle cell disease show a decrease in cholesterol and fractions, apo-A1 and Apo-B levels, and increased LDL oxidation. Despite the small number of patients, it was shown, for the first time, that PON-1 arylesterase activity is decreased in this disease. Moreover, the decrease correlated with serum transferrin and ferritin levels. Both transferrin and ferritin were the main predictors of arylesterase and paraoxonase activities in the patients with sickle cell disease. No differences were observed with PON-1 L55M, and PON-2 and PON-3 polymorphisms. RR PON-1 Q192R polymorphism is likely to be a protective factor against oxidative damage, as shown by the higher paraoxonase activities, and lower serum ferritin and iron levels and transferrin saturation observed in those patients. Nevertheless, further research is needed to better understand the relationship between cholesterol metabolism, iron metabolism, and PON-1 in sickle cell disease.

## Figures and Tables

**Table 1:** Sequences of primers to determine PON1 and PON2 polymorphisms.

**PON-1 Primers**	**Sequences 5′→3′**
PON-1 55F	GAG TGA TGT ATA GCC CCA GTT TC
PON-1 55R	AGT CCA TTA GGC AGT ATC TCCg
PON-1 192F	TTG AAT GAT GTT GCT GTG GGA CCT GAG
PON-1 192R	CGA CCA CGC TAA ACC CAA ATA CAT CTC CCA GaA
**PON-2 Primers**	**Sequences 5′→3′**
PON-2 148F	AGT GGA AAT TTT TAA ATT TGA AGC AG
PON-2 148R	TTG TTT GCA AAT GCT GGG GAT
PON-2 311F	GGT TCT CCG CAT CCA GAA CAT TgaA
PON-2 311R	TGT TAA GaT ATC GCA TCA TGC C

**Table 2:** Gender and age of patients with sickle cell disease and healthy controls.

Gender	Groups		
	Sickle Cell Disease (n = 43)	Healthy Control (n = 43)	*P-*Value ^(1)^
Female	30 (69.77%)	30 (69.77%)	1.0
Male	13 (30.23%)	13 (30.23%)	1.0
Age (years)	38.1 ± 11.72	37.8 ± 11.32	0.904

^(1)^ Chi-square test (χ^2^) and Student’s t-test.

**Table 3:** Lipid profile and PON-1 activities in patients with sickle cell disease and healthy controls.

Parameters	Groups						
	Sickle Cell Disease (n = 43)		Healthy Controls (n = 43)		*P* Value ^(1)^	*R* Value ^(2)^	*R* Value ^(3)^
	Mean ± SD	Min−Max	Mean ± SD	Min−Max			
Total cholesterol (mg/dL)	132.51 ± 27.93	78−197	181.44 ± 35.99	104−277	0.01	0.407	0.098
HDL-C (mg/dL)	36.16 ± 11.38	14−66	50.65 ± 13.17	27−82	0.01	0.334	0.296
LDL-C (mg/dL)	71.18 ± 23.78	29−126	104.93 ± 32.23	44−208	0.01	0.270	0.060
VLDL-C (mg/dL)	25.27 ± 9.44	11−56	25.4 ± 10.06	11−56	>0.05	0.131	−0.215
Triglycerides (mg/dL)	125.81 ± 47.14	57−278	129.07 ± 65.04	56−373	>0.05	0.111	−0.227
Apolipoprotein A1	107.78 ± 21.21	75.5−164.2	162.0 ± 26.6	113.3−225	0.01	0.427	0.259
Apolipoprotein B	65.13 ± 20.3	33.8−107.9	89.03 ± 22.32	53.6−161.5	0.01	0.170	−0.102
ApoB:ApoAI ratio	0.62 ± 0.20	0.21−1.07	0.56 ± 0.16	0.31−1.14	0.08	−0.092	0.330
TG:HDL-C ratio	3.99 ± 2.54	0.9−15.1	2.85 ± 1.93	0.8−10.4	0.006	−0.120	0.034
LDL-C:HDL-C ratio	2.10 ± 0.80	0.5−4.8	2.17 ± 0.82	0.8−4.7	>0.05	−0.144	−0.340
C−reactive protein(mg/L)	7.7 ± 9.3	0.8−59.4	2.69 ± 3.32	0.2−14.7	0.001	−0.179	−0.229
Anti−oxLDL (U/mL)	2.84 ± 1.52	0.91−10.33	1.74 ± 0.74	0.61−4.5	0.001	−0.107	−0.086
Paraoxonase−1 activities							
Paraoxonase (U/mL)	80.3 ± 45.8	12−184	100.1 ± 55.2	24−232	0.078	-	-
Arylesterase (U/mL)	69.9 ± 20.3	24.5−122	89.7 ± 27.3	1−161	0.001	-	-

^(1)^ Mean comparison by Student’s t-test or Mann–Whitney U test between the groups; ^(2)^ Pearson correlation coefficient between parameters and arylesterase activity in sickle cell disease group; ^(3)^ Pearson correlation coefficient between parameters and paraoxonase activity in sickle cell disease group; HDL-C: high-density lipoprotein-cholesterol; LDL-C: low-density lipoprotein-cholesterol; TG: triglycerides; VLDL-C: very low-density lipoprotein cholesterol; SD: standard deviation.

**Table 4:** Relationship between biochemical parameters and PON-1 arylesterase and paraoxonase activities.

Sickle Cell Disease (n = 43)				Arylesterase Activity				Paraoxonase Activity			
Parameters	Mean ± SD	Min−Max	RV ^(1)^	β Value	Standard Error	*P* Value	Model ^(1)^	β value	Standard Error	*P* Value	Model ^(2)^
Lactate dehydrogenase (U/L)	1162 ± 426.78	589−2326	240−480	−0.237	0.009	−0.229	R^2^ = 0.011; *P* = 0.513	−0.146	−0.02	−0.449	R^2^ = 0.020; *P* = 0.381
Total bilirubin (mg/dL)	4.23 ± 2.58	1.13−13.8	0.20−1.00	6.230	50.53	0.343	R^2^ = 0.015; *P* = 0.748	−7.057	110.04	0.277	R^2^ = 0.022; *P* = 0.652
Indirect bilirubin (mg/dL)	2.98 ± 1.68	0.66−7.92	0.10−0.60	−3.904	50.32	0.359	R^2^ = 0.048; *P* = 0.603	4.738	109.58	0.261	R^2^ = 0.071; *P* = 0.432
Direct bilirubin (mg/dL)	1.24 ± 1.63	0.3−9.67	<0.30	−4.134	50.71	0.320	R^2^ = 0.074; *P* = 0.582	4.260	110.44	0.300	R^2^ = 0.098; *P* = 0.429
Hemoglobin (g/dL)	8.27 ± 1.03	6.2−10.6	12.0−16.0	0.052	10.91	0.925	R^2^ = 0.001; *P* = 0.878	−0.902	23.71	0.109	R^2^ = 0.002; *P* = 0.772
Hematocrit (%)	23.53 ± 3.02	17.6−31.25	35−47	−0.187	3.58	0.725	R^2^ = 0.014; *P* = 0.758	0.728	7.78	0.176	R^2^ = 0.013; *P* = 0.779
Reticulocyte (%)	12.08 ± 6.03	4.06−35.02	0.5−2.7	−0.031	0.655	0.870	R^2^ = 0.049; *P* = 0.599	−0.295	1.42	0.129	R^2^ = 0.135; *P* = 0.144
Leucocytes (×10^3^/mm^3^)	10.73 ± 3.45	6.33−21.4	4−11	−0.394	1.03	0.029	R^2^ = 0.168; *P* = 0.146	−0.224	2.24	0.204	R^2^ = 0.173; *P* = 0.135
Ferritin (ng/dL)	568.36 ± 917.94	56−4872	15−150	0.451	0.015	0.227	R^2^ = 0.047; *P* = 0.277	0.252	0.032	0.166	R^2^ = 0.000; *P* = 0.923
Transferrin (mg/dL)	231.17 ± 42.97	150−335	250−380	0.507	0.091	0.018	R^2^ = 0.250; *P* = 0.032	0.633	0.273	0.003	R^2^ = 0.316; *P* = 0.011
Transferrin saturation (%)	45.11 ± 18.32	11.09−91.9	20−40	−0.415	1.226	0.739	R^2^ = 0.266; *P* = 0.064	0.064	2.590	0.959	R^2^ = 0.316; *P* = 0.030
Iron (µg/dL)	120 ± 49.54	41−244	37−145	0.580	0.454	0.482	R^2^ = 0.332; *P* = 0.055	−0.520	0.960	0.966	R^2^ = 0.378; *P* = 0.028
TIBC (µg/dL)	274.88 ± 62.58	178−489	228−428	−0.734	0.202	0.298	R^2^ = 0.367; *P* = 0.069	−0.335	0.427	0.626	R^2^ = 0.385; *P* = 0.054
Fibrinogen (mg/dL)	350.72 ± 108.9	178−712	150−400	−0.30	0.031	0.856	R^2^ = 0.063; *P* = 0.272	−0.002	0.991	0.340	R^2^ = 0.053; *P* = 0.340

^(1)^ Multivariate linear regression by arylesterase activity; ^(2)^ Multivariate linear regression by paraoxonase activity; RV: reference value: TIBC: Total Iron Binding Capacity; SD: standard deviation 3.6 PON-1, PON-2, and PON-3 polymorphisms.

**Table 5:** Distribution of genotypes and allelic frequency of PON-1 192QR and 55LM polymorphisms, PON-2 148AG and 311SC polymorphisms, and PON-3 10340GT, 2115AT, 45486AC, and 55146CT polymorphisms in patients with sickle cell disease and healthy controls.

Polymorphisms	Groups		
	Sickle Cell Disease (*n* = 43)	Healthy Control (*n* = 43)	*P-*Value ^(1)^
**PON-1 Q192R**			
QQ	10 (23.2)	24 (55.8)	0.0064
QR	19 (44.2)	13 (30.2)	
RR	14 (30.6)	6 (13.9)	
Q allele	20 (45)	30 (71)	
R allele	24 (55)	13 (29)	
**PON-1 L55M**			
LL	21 (48.8)	20 (46.5)	>0.05
LM	10 (23.2)	14 (34.8)	
MM	12 (27.9)	9 (18.6)	
L allele	26 (60)	28 (64)	
M allele	17 (40)	15 (36)	
**PON-2 A148G**			
AA	24 (55.8)	30 (69.7)	0.178
AG	12 (27.9)	11(25.6)	
GG	7 (16.3)	2 (4.6)	
A allele	30 (70)	36 (83)	
G allele	13 (30)	7 (17)	
**PON-2 S311C**			
SS	23 (53.4)	23 (53.4)	0.385
SC	16 (37.2)	12 (27.9)	
CC	4 (9.3)	8 (18.6)	
S allele	31 (72)	29 (67)	
C allele	12 (28)	14 (33)	
**PON-3 G10340T**			
GG	1 (2.3)	2 (4.6)	0.621
GT	14 (32.5)	17 (39.5)	
TT	28 (65.1)	24 (55.8)	
G allele	8 (19)	10 (24)	
T allele	35 (81)	33 (76)	
**PON-3 A2115T**			
AA	0 (0)	1 (2.3)	0.713
AT	3 (6.9)	4 (9.3)	
TT	40 (93)	38 (88.3)	
A allele	1 (3)	3 (7)	
T allele	42 (97)	40 (93)	
**PON-3 A45486C**			
AA	0 (0)	0 (0)	0.676
AC	2 (4.6)	4 (9.3)	
CC	41 (95.3)	39 (90.6)	
A allele	1 (2)	2 (5)	
C allele	42 (98)	41 (95)	
**PON-3 C55146T**			
CC	41 (95.3)	38 (88.4)	0.433
CT	2 (4.6)	5 (11.6)	
TT	0 (0)	0 (0)	
C allele	42 (98)	40 (94)	
T allele	1 (2)	3 (6)	

Results are expressed as n (%); ^(1)^ *P* value = Chi-square test (χ^2^) or Fisher’s exact test.
